# BK Virus-Hemorrhagic Cystitis Following Allogeneic Stem Cell Transplantation: Clinical Characteristics and Utility of Leflunomide Treatment

**DOI:** 10.4274/tjh.2015.0131

**Published:** 2016-08-19

**Authors:** Young Hoon Park, Joo Han Lim, Hyeon Gyu Yi, Moon Hee Lee, Chul Soo Kim

**Affiliations:** 1 Inha University Faculty of Medicine and Hospital, Department of Hematology-Oncology, Incheon, Republic of Korea

**Keywords:** BK virus, hemorrhagic cystitis, allogeneic stem cell transplantation, Leflunomide

## Abstract

**Objective:**

BK virus-hemorrhagic cystitis (BKV-HC) is a potential cause of morbidity and mortality in patients having undergone allogeneic stem cell transplantation (Allo-SCT). We analyzed the clinical features of BKV-HC following Allo-SCT and reported the utility of leflunomide therapy for BKV-HC.

**Materials and Methods:**

From January 2005 to June 2014, among the 69 patients that underwent Allo-SCT in our institution, the patients who experienced BKV-HC were investigated retrospectively.

**Results:**

HC was observed in 30 patients (43.5%), and among them, 18 of the cases (26.1%) were identified as BKV-HC. The median age of the patients (12 males and 6 females) was 45 years (minimum-maximum: 13-63). Patients received Allo-SCT for acute myeloid leukemia (n=11), aplastic anemia (n=4), myelodysplastic syndrome (n=2), and non-Hodgkin lymphoma (n=1). The donor types were human leukocyte antigen (HLA)-matched sibling donor for six patients, HLA-matched unrelated donor for nine, and haploidentical familial donor for two. The median onset and duration of BKV-HC was on day 21 after transplantation (minimum-maximum: 7-97) and 22 days (minimum-maximum: 6-107). Eleven patients (62.1%) had grade I-II HC and seven patients (38.9%) had grade III-IV (high-grade) HC. Among the seven patients who had high-grade HC, one had complete response, one had partial response, and five had no response. Among the five nonresponders, one died of BKV-HC associated complications. The remaining four patients were treated with leflunomide, achieving complete response (n=2) and partial response (n=2). The median duration from the start of leflunomide therapy to response was 13 days (minimum-maximum: 8-17 days). All patients tolerated the leflunomide treatment well, with three patients having mild gastrointestinal symptoms, including anorexia and abdominal bloating.

**Conclusion:**

BKV-HC was commonly observed in patients with HC following Allo-SCT. In high-grade BKV-HC patients who do not respond to supportive care, leflunomide may be a feasible option without significant toxicity.

## Abstract (tr)

**Amaç:**

BK-virüs hemorajik sistiti (BKV-HS) allojenik kök hücre nakli (Allo-KHN) uygulanan hastalarda morbidite ve mortalitenin önemli bir nedenidir. Bu çalışmada Allo-KHN sonrası BKV-HS olan olguların klinik özellikleri ve leflunomid tedavisinin BKV-HS’deki etkinliği araştırılmıştır.

**Gereç ve Yöntemler:**

Kliniğimizde Ocak 2005-Haziran 2014 arası Allo-KHN uygulanmış 69 hastada, BKV-HS geçirmiş olanlar retrospektif olarak değerlendirildi.

**Bulgular:**

Otuz hastada (%43,5) HS gözlendi. Bu olguların 18’inde (%26,1) BKV-HS’si saptandı. Hastaların (12’si erkek, altısı kadın) medyan yaşı 45 (13-63) idi. Hastalara akut miyeloid lösemi (n=11), aplastik anemi (n=4), miyelodisplastik sendrom (n=2) ve non-Hodgkin lenfoma (n=1) nedeni ile Allo-KHN uygulanmıştı. Altısında insan lökosit antijeni (İLA)-uygun kardeş, dokuzunda İLA-uygun akraba dışı donör ve ikisinde haplo-identik donör kullanılmıştı. Transplant sonrası BKV-HS medyan başlangıç zamanı 21 gün (7-97 gün), medyan süresi 22 gün (6-107 gün) idi. On bir olguda (%62,1) derece I-II, yedi olguda (%38,9) derece III-IV (yüksek derecede) HS saptandı. Yüksek derece HS’li yedi hastanın, birinde tam yanıt, birinde kısmi yanıt elde edilirken, beş hastada yanıt alınamadı. Yanıt alınmayan beş hastanın birisi BKV-HS ilişkili komplikasyonlardan kaybedildi. Geri kalan dört hasta leflunomid ile tedavi edildi. Bu hastaların ikisinde tam yanıt, ikisinde kısmi yanıt elde edildi. Leflunomidin başlangıcından itibaren medyan yanıt süresi 13 gündü (8-17 gün). Tüm hastalar leflunomidi iyi tolere ederken, üç hastada anoreksi ve abdominal gaz şikayetleri dahil hafif şiddetli gastrointestinal yan etkiler gözlendi.

**Sonuç:**

Allo-KHN sonrası izlemde BKV-HS yaygın olarak gözlenmiştir. Destek tedavisine yanıt vermeyen yüksek derece BKV-HS’li olgularda leflunomid, anlamlı toksisitesi olmaksızın bir seçenek olabilir.

## INTRODUCTION

Hemorrhagic cystitis (HC) is a potential cause of morbidity and mortality in patients that have undergone allogeneic stem cell transplantation (Allo-SCT) [1,2,3]. Its incidence ranges from 5% to 68% of Allo-SCT recipients, with severe-grade hematuria in 29%-44% of cases [3,4,5,6,7]. Variable etiologies for the development of HC in Allo-SCT recipients include noninfectious and infectious causes. As an infectious cause of HC, BK virus-HC (BKV-HC) occurs later after transplantation, usually in the post-engraftment period [3]. The BKV, a member of the family Polyomaviridae, is typically acquired in childhood and embedded in urothelial cells of the urinary tract in the latent dormant stage [8]. BKV reactivation is commonly associated with HC in Allo-SCT settings, occurring in 10% to 25% of patients [8]. The clinical symptoms of BKV-HC vary to a great extent in Allo-SCT recipients from asymptomatic hematuria to massive hemorrhage leading to urinary obstruction and renal failure [2,9,10]. Previous studies demonstrated that BKV-HC is associated with not only increased morbidity and but also increased mortality in Allo-SCT patients [6,7,11,12], and studies have also defined potential risk factors for the development of BKV-HC [4,5,13,14,15], most of which have not been observed consistently in several reports.

Leflunomide, an immunomodulatory agent with antiviral activity, has been found effective against cytomegalovirus (CMV), herpes simplex, and BKV based on in vitro data [6,16,17]. In renal allografts, leflunomide has been widely used to treat biopsy-proven BKV nephropathy [18,19], but it has not been well studied in Allo-SCT settings. Only two reports showed satisfactory results of leflunomide therapy in the treatment of BKV-HC after Allo-SCT [20,21].

In this retrospective study, we report the incidence, severity, and outcome of clinical BKV-HC in patients who underwent Allo-SCT to treat variable hematologic diseases. Furthermore, we report high-grade BKV-HC patients who achieved favorable response to leflunomide therapy.

## MATERIALS AND METHODS

### Patients

A total of 69 patients underwent Allo-SCT in our institution from January 2005, when BKV polymerase chain reaction (PCR) testing became clinically available, to June 2014. Baseline demographic and transplantation data were collected, including age, sex, underlying disease, conditioning regimen, stem cell source, donor type, prophylaxis to infection and graft-versus-host disease (GVHD), time to engraftment, presence and grade of GVHD, and survival at last follow-up. Patients received either myeloablative or reduced-intensity conditioning regimen according to disease status, age, or comorbidities. As prophylaxis against HC, hyperhydration (intravenous isotonic saline over 3 L/m2 per day) with forced diuresis was used to prevent toxicity of conditioning regimen for Allo-SCT. In addition, for patients receiving a preparative regimen containing cyclophosphamide (Cy), 2-mercaptoethane sulfonate (MESNA) was given prior to the Cy administration and thereafter as a continuous infusion until the last dose of Cy. Acute GVHD and chronic GVHD were diagnosed and graded according to previously published criteria [22,23].

### Diagnosis of BK Virus-Hemorrhagic Cystitis

HC was defined as the presence of sustained hematuria and urinary symptoms after the beginning of conditioning therapy in the absence of gynecological-related bleeding, generalized bleeding diathesis, and urinary tract infection. Severity of HC was graded according to the following criteria [7]: grade 0 (no hematuria), grade I (microscopic hematuria), grade II (macroscopic hematuria), grade III (macroscopic hematuria with presence of blood clots), and grade IV (macroscopic hematuria with clots and renal impairment due to urinary obstruction). Grades III and IV were defined as high-grade HC. The date of onset of HC was defined as the first day of symptoms or laboratory evidence appearing after transplant. Routine urinalysis was performed at least twice a week during hospitalization and thereafter at outpatient visits. For patients who had urinary symptoms or gross hematuria, BKV testing by a qualitative PCR-based method was performed with the urine specimen to identify the presence of virus.

### BK Virus Polymerase Chain Reaction Test

Each urine sample was submitted to DNA extraction using a QIAamp DNA Mini Kit (QIAGEN, Hilden, Germany) according to the manufacturer’s instructions. A primer was designed for a highly conserved region of gene large T antigen to obtain a 160-bp amplicon from the BKV genome using the GeneAmp 2720 Thermal Cycler (ABI, Foster City, CA, USA). PCR of urine for BKV viruria had a sensitivity of 90% and a specificity of 96.5% (cut-off value: 160 copies/mL).

### Urine Cytology

Each urine specimen was processed for cytological evaluation by centrifugation. After removal of the supernatant, the sediment was examined for clarity. Cytospin slides were fixed in 95% alcohol, stained by the Papanicolaou method, and observed for the presence of urine decoy cells (characterized by a ground-glass appearance with an enlarged nucleus, which is occupied by a homogeneous basophilic inclusion surrounded by chromatin) by a well-trained pathologist.

### Treatment and Criteria of Response

All patients diagnosed with BKV-HC received supportive treatment, including hyperhydration with normal saline, forced diuresis, urine alkalinization, analgesics, and blood transfusion to maintain platelet counts at ≥50x109/L and a hemoglobin level of ≥8 g/dL. Foley catheterization and bladder irrigation were considered for patients with high-grade HC who had no response (NR) to supportive care. Leflunomide therapy was indicated as follows: 1) grade III-IV HC; 2) no change or worsening in urinary symptoms or grade of hematuria during supportive care within 2 weeks; 3) no abnormality in liver function tests; 4) absolute neutrophil count of >1.0x109/L; and 5) no abnormality on chest radiology. For the patients receiving leflunomide therapy, leflunomide at 100 mg/day orally was used as a loading dose for 5 days, followed by maintenance doses of 20 mg/day until resolution of hematuria and urinary symptoms. Clinical response was defined as follows: complete response (CR), completely improved in symptoms with absence of hematuria; partial response (PR), downgrading of severity with persistent hematuria; NR, unchanged or worsening urinary symptoms or grade of hematuria; refractoriness, NR even after about 2 weeks of supportive care. The response was evaluated after 20 days of leflunomide treatment.

### Statistical Analysis

The cumulative incidence of BKV-HC was estimated with the interval starting at Allo-SCT until the day of the first PCR-positive urine sample. The Mann-Whitney test and chi-square test were used for comparisons for continuous and categorical data, respectively, between the patients with low-grade BKV-HC and those with high-grade BKV-HC. Univariate and multivariate analyses of risk factors for BKV-HC occurrence were performed using the Cox proportional hazard model. SPSS 14.0 (SPSS Inc., Chicago, IL, USA) was used for all statistical analyses, and all were two-sided. Statistical significance was defined as p<0.05. This study was approved by the Institutional Review Board of the Inha University Hospital.

## RESULTS

### Patient Characteristics and Transplant Outcome

The demographic characteristics of the HC patients are presented in Table 1. In our study, a total of 30 of 69 (43.5%) patients who underwent Allo-SCT developed HC. Of these, 18 (26.1%) were diagnosed with BKV-HC and were included in this study. Seven (38.9%) patients received antithymoglobulin for in vivo T-cell depletion. Nine patients (50%) had acute GVHD, and two patients (11.1%) had grade III-IV GVHD. These two patients received systemic glucocorticoid therapy (1-2 mg/kg prednisone) without additional immunosuppressant agents and showed a good response to steroid therapy. Twelve patients (66.7%) had chronic GVHD, and seven patients (38.9%) showed extensive chronic GVHD. Among the seven patients with extensive GVHD, two patients showed corticosteroid-refractory chronic GVHD and were treated with mycophenolate mofetil, but failed to respond.

### Clinical Features of BK Virus-Hemorrhagic Cystitis

The clinical characteristics of BKV-HC are detailed in Table 2. Eleven patients (62.1%) had low-grade HC and seven patients (38.9%) had high-grade HC. The median onset and duration of BKV-HC was on day 21 (minimum-maximum: 7-97) after transplantation and 22 days (minimum-maximum: 6-107), respectively. There was no significant difference between the onset in patients with low-grade HC or high-grade HC (21 vs. 21 days, p=0.633). In two patients (11.1%), BKV-HC developed before neutrophil engraftment. Nine (50.0%) patients had concomitant cytomegalovirus viremia, and among them, four patients received ganciclovir treatment. Of patients with acute GVHD (n=9), seven (77.7%) developed acute GVHD after onset of BKV-HC and, conversely, two (22.3%) developed BKV-HC after the diagnosis of acute GVHD. Four (22%) patients had urinary cytologic changes compatible with BKV.

### Treatment

The majority of the patients received supportive treatment, including intravenous hydration and/or blood transfusion (Table 2). A urinary catheter was inserted for three patients with HC of grade III and two patients with HC of grade IV. We slightly tapered the immunosuppressant by monitoring the serum trough level in low-grade BKV-HC patients with no evidence of GVHD or grade I-II GVHD. In contrast, in the patients with high-grade BKV-HC, reduction or withdrawal of the immunosuppressant was not done as a treatment option for BKV-HC due to the risk of GVHD. After supportive care, nine patients (50.0%) achieved CR, four patients (22.2%) PR, and five patients (27.8%) NR. All patients with low-grade HC showed better responses than PR to supportive treatment. Out of the seven patients who had high-grade HC, one had CR, one PR, and five NR. Response (CR+PR) rate in patients with low-grade HC was significantly higher than in those with high-grade HC (100% vs. 28.6%, p=0.004). Among the five nonresponders, one died of BKV-HC-associated renal failure with no evidence of progression of underlying disease. The remaining four patients were treated with oral leflunomide as salvage therapy.

### Risk Factors for BK Virus-Hemorrhagic Cystitis Occurrence

In univariate analysis, HLA mismatching and acute GVHD grades II-IV were associated with a higher risk of developing BKV-HC (p=0.034 and p<0.001, respectively). In multivariate analysis, only acute GVHD grades II-IV were independent risk factors for BKV-HC (hazard ratio 3.26, 95% CI 1.42-13.5, p=0.002) in this study (Table 3).

### Leflunomide Therapy

Detailed information about the four patients receiving leflunomide therapy is summarized in Table 4. Of the four patients, three had acute myeloid leukemia and one had high-risk myelodysplastic syndrome. All patients had acute GVHD before development of BKV-HC. After leflunomide treatment, two (50%) patients achieved CR and two (50%) achieved PR. One CR patient and one PR patient had a negative PCR for BKV, but BKV in urine still remained detectable in one patient achieving CR. The median duration from the start of leflunomide therapy to response was 13 days (minimum-maximum: 8-17). The dose of leflunomide was not reduced for any patients. All patients tolerated the leflunomide treatment well, with three patients having mild gastrointestinal symptoms, including anorexia and abdominal bloating. No significant adverse effects, such as hepatotoxicity, skin reactions, diarrhea, bone marrow suppression, or pneumonia, were observed during leflunomide treatment. There was no recurrence of hematuria in the two patients achieving CR after discontinuation of leflunomide therapy. Of the patients achieving PR, one died of leukemia relapse 16.3 months after Allo-SCT. The remaining three patients are still alive without hematuria.

## DISCUSSION

Our results, with an overall incidence of HC following Allo-SCT of 43.5%, are consistent with other studies’ findings, which reported frequencies of HC following SCT ranging from 5% to 68% [3,4,5,6,7]. BKV was identified in 60.0% of cases (18 of 30 patients) by a qualitative PCR-based assay. A study of 22 Allo-SCT patients who experienced HC showed that the most frequent virus detected was BKV, with an incidence of 54.5% of patients, followed by JC virus and CMV [24]. In a study of 102 children who underwent Allo-SCT for malignancies and nonmalignant diseases, HC occurred in 26 patients (25.5%), and among them, BKV was identified in 21 (80.8%) patients [25]. These findings demonstrated that HC is a frequent complication after Allo-SCT and BKV is mainly responsible for HC in Allo-SCT recipients.

Hemorrhagic cystitis after SCT frequently caused prolongation of hospitalization and occasionally death [10,24,26]. Gilis et al. demonstrated that the median duration of hospitalization for Allo-SCT was significantly longer for patients developing BKV-HC compared with those without BKV-HC (50 vs. 40 days, p<0.001) [26]. In addition, in a series of 12 patients with BKV-HC, HC-associated renal failure was the main cause of death in two (16.7%) patients, who failed to respond to any treatment including administration of intravenous cidofovir [24]. In our study, among the seven high-grade BKV-HC patients, one died of BKV-HC-related renal failure. These observations suggest that prompt alternative treatment should be considered in patients with BKV-HC, and especially high-grade HC, who had refractoriness to initial supportive treatment.

Currently, there is no established antiviral drug for the treatment of BKV-HC, although reduction in immunosuppression is necessary for clearance of the BKV. Salvage treatment with antiviral agents, including cidofovir, leflunomide, and fluoroquinolones, has been considered in renal transplant recipients when reduction of immunosuppression alone could not improve renal function, with no antiviral agents shown to be effective in randomized controlled studies [18,27,28,29]. In Allo-SCT settings, prior reports showed that treatment with varying doses of cidofovir, having activity against CMV, adenovirus, and polyomaviruses, was feasible [30,31,32]. However, the potential nephrotoxicity of cidofovir, resulting in direct tubular toxicity, still remains a major obstacle to its use. Leflunomide, an immunomodulatory agent, has modest antipolyomavirus activity in vitro, although the precise mechanism of action remains to be fully understood [6,17]. In renal transplant patients, leflunomide has been used to treat BKV nephropathy, resulting in a significant decline in the BK viral load with relatively stable renal function without the toxicity of leflunomide [10,18]. Thus far, there are limited clinical data on the utility of leflunomide therapy in the treatment of BKV-HC patients in Allo-SCT settings. In a series of 14 adult patients with BKV-HC undergoing Allo-SCT, leflunomide therapy showed favorable results for BKV-HC treatment with no serious adverse effects, with CR and PR being seen in 50% and 37.5% of patients, respectively [21]. In a pilot study with five pediatric patients with severe BKV-HC after Allo-SCT, significantly shorter duration of BKV-HC (p<0.01) and decreased BK viral loads in blood (p<0.01) and urine (p=0.03) were observed after leflunomide therapy compared with historical controls [20]. In this study, CR was achieved in 50% of the patients receiving leflunomide therapy. In addition, potential adverse effects associated with leflunomide treatment, including hepatotoxicity and bone marrow suppression, were not observed in any treated patients. Our results were similar to previous reports regarding leflunomide therapy in adult patients. These data suggest that leflunomide is effective in the treatment of posttransplant high-grade BKV-HC, especially in cases refractory to supportive care.

This study has potential limitations, mostly stemming from its small sample size and retrospective design. Several studies have indicated that BK viruria was quantitatively related to the occurrence of HC after Allo-SCT and clinical response to antiviral therapy may be linked with reduction of viral loads in blood and/or urine [20,21,26,33]. However, we performed only a qualitative test in urine samples, but BKV was still detectable in one patient achieving CR after leflunomide therapy, suggesting that complete clearance of the virus in urine may not be necessary in BKV-HC patients treated with leflunomide.

In summary, BKV-HC is a common complication of Allo-SCT and is associated with significant morbidity, especially in high-grade BKV-HC. In high-grade BKV-HC patients who fail to respond to supportive care, leflunomide therapy may be a feasible treatment option without significant toxicity. Prospective randomized controlled trials are warranted to evaluate the efficacy and safety of leflunomide in the treatment of BKV-HC after Allo-SCT.

## Ethics

Ethics Committee Approval: This study was approved by the Institutional Review Board of the Inha University Hospital; Informed Consent: It was taken.

## Figures and Tables

**Table 1:**
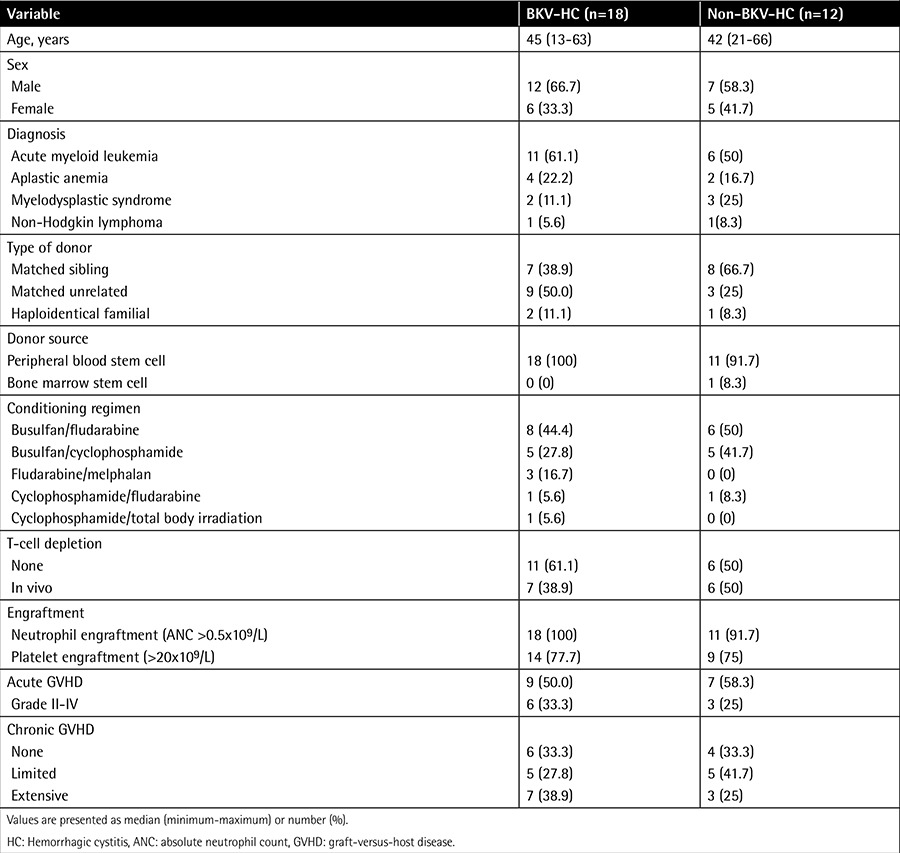
Demographic characteristics of the hemorrhagic cystitis patients and posttransplant outcomes.

**Table 2:**
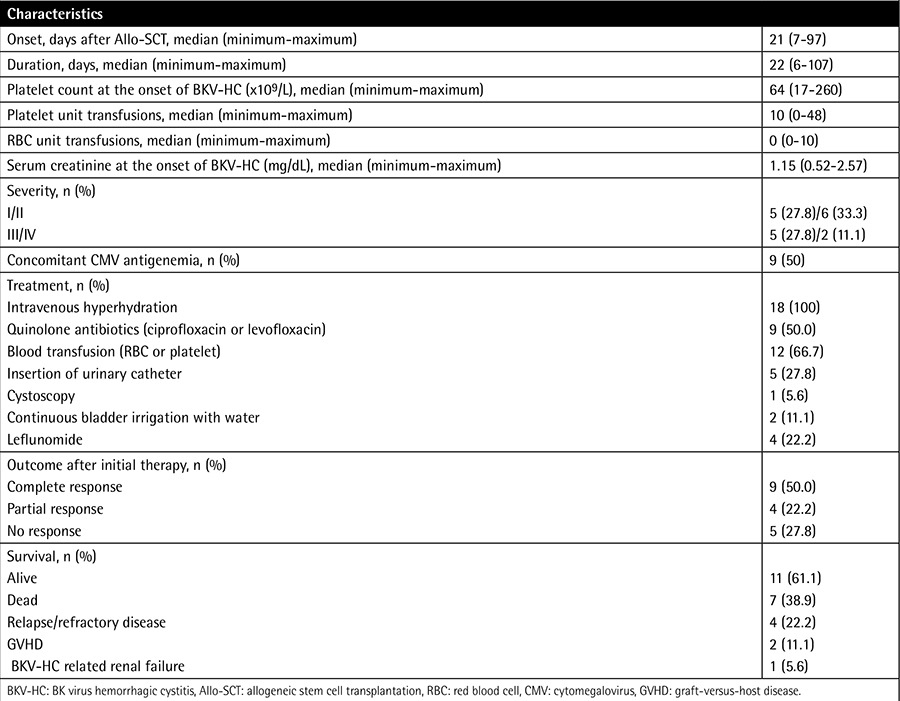
Characteristics of BK virus-hemorrhagic cystitis and treatment outcomes.

**Table 3:**
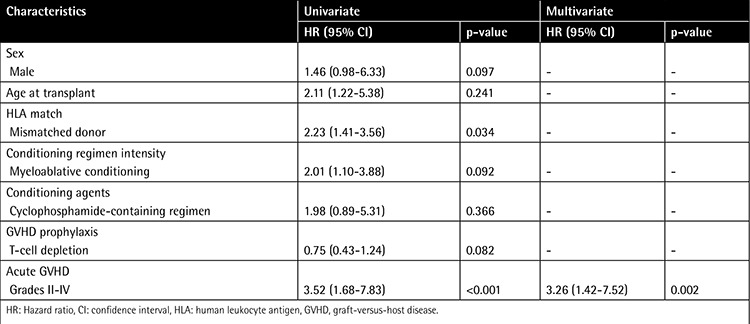
Univariate and multivariate analysis for development of BK virus hemorrhagic cystitis.

**Table 4:**
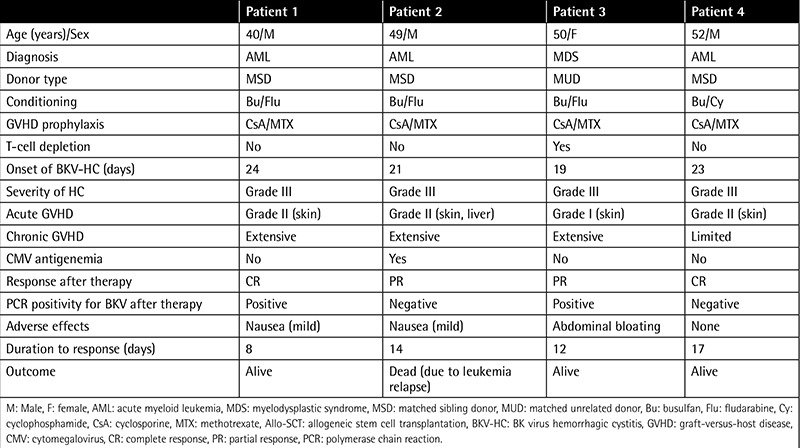
Clinical characteristics of the patients receiving leflunomide therapy.
